# Cutaneous polyarteriitis nodosa with Grave’s disease and cholestatic liver disease

**DOI:** 10.1186/1546-0096-9-S1-P90

**Published:** 2011-09-14

**Authors:** V Vassileva, B Varbanova

**Affiliations:** 1 Medical University of Varna, Department of Paediatrics, Varna, Bulgaria

## 

Cutaneous polyarteriitis nodosa (CPAN) is a rare relapsing disease in childhood. Associations with other autoimmune diseases have been described in some patients. The etiologic role of Streptococcal infection, HCV, HBV and other triggers has been widely discussed in the past with no conclusive data about their exact mechanism of action and clinical significance.

We present the 10-year follow-up of a girl with relapsing CPAN who developed further clinical manifestations of autoimmune diseases and conditions. At the age of 12 she was admitted with high fever, polymorphic rash, severe myalgia and hyperesthesia, arthritis, livedo reticularis and painful subcutaneous nodules on the legs. Mononeuritis multiplex was found by EMG and the histological examination confirmed the diagnosis. Elevated ASO, positive serology for HCV with normal liver enzymes and negative ANCA were also established. The remission was achieved with corticosteroids and lasted for five years. As the antibiotic treatment did not lead to the normalization of ASO, tonsillectomy was performed at the end of the first year. Negative tests for HCV (incl. PCR) were registered at that time. Two years after the discontinuation of the therapy, at the debut of a mild flare, presenting with fever, myalgia and single erythematous eruptions on the legs, she had developed severe symptoms of hyperthyroidism. After the exclusion of Hashimoto thyroiditis, Grave’s disease was diagnosed and a treatment with propylthiouracil was initiated. On the third day she developed devastating necrotic vasculitis. ANCA remained negative. The remission was achieved with corticosteroids and cyclophosphamide. After a 3-year free-of-disease period, a mild cholestatic disease, not consistent with current infection or treatment was established. The last flare occurred recently, manifested with fever, myalgia, arthralgia, modest rash and mononeuritis multiplex. Histological data reaffirmed the diagnosis of PAN. The treatment with corticosteroids and methotrexate improved all symptoms, including those of the liver disease.

During the 10 –year follow-up of this patient, we did not find the typical systemic organ involvement corresponding to PAN, but observed overlapping autoimmune diseases and conditions. The latter associations could be regarded in the context of the former HCV and streptococcal infections.

